# Proceedings of the Buffalo Medical and Surgical Association

**Published:** 1890-06

**Authors:** W. H. Bergtold

**Affiliations:** Secretary


### PROCEEDINGS OF THE BUFFALO MEDICAL AND SURGICAL ASSOCIATION.

Reported by W. H. BERGTOLD, M. D., *Secretary.*

REGULAR meeting, held May 6th, at 8.45 P. M., at No. 14 West Mohawk street.

The president, Dr. A. A. HUBBELL, in the chair.

In the absence of Dr. Bergtold, Dr. J. Falk acted as secretary.

Dr. E. N. PFOHL was elected a member.

Dr. HERMAN MYNTER presented a patient on whom he had made a resection of the elbow for tubercular arthritis.

Dr. GEO. E. FELL read a paper on The Liver in Health and Disease, and was followed by Dr. W. H. HEATH, who spoke on Syphilis.

### DISCUSSION.

Dr. MYNTER believes in the curability of syphilis, as he had seen a case, which was declared to be specific by an eminent professor of Copenhagen, come to him five years later with a genuine chancre.

Dr. HARTWIG does not believe in the curability of syphilis. Dr. Hartwig asked Dr. Heath whether he had ever seen a case of syphilis hereditaria tarda, so-called. He had lately had a case in the person of a boy eleven years old. Dr. Hartwig also spoke of the use of chromic acid in treating mucous patches.

